# Effect of fish paste products, fish balls ‘tsumire’, intake in Sprague–Dawley rats

**DOI:** 10.1017/jns.2021.57

**Published:** 2021-08-13

**Authors:** Kazunari Kadokura, Tsuyoshi Tomita, Kohei Suruga

**Affiliations:** Research & Development Division, Products Development Department, Kibun Foods Inc., 86 Yanokuchi, Inagi, Tokyo 206-0812, Japan

**Keywords:** Fish paste products, Fish balls, Tsumire, Liver function, Rats, ALA, α-linolenic acid, ALB, albumin, ALP, alkaline phosphatase, ALT, alanine transaminase, AMY, amylase, APP, Alaska pollack protein, AST, aspartate aminotransferase, BCAAs, branched-chain amino acids, BChE, butyrylcholinesterase, BUN, blood urea nitrogen, ChE, cholinesterase, CHO, cholesterol, CRE, creatinine, D-BIL, direct bilirubin, DHA, docosahexaenoic acid, EPA, eicosapentaenoic acid, F-CHO, free cholesterol, GLP-1, glucagon-like peptide-1, GLU, glucose, HDL-C, high-density lipoprotein cholesterol, IP, inorganic phosphorus, LAP, leucine aminopeptidase, LDL-C, low-density lipoprotein cholesterol, TAG, triacylglycerol, TBA, total bile acids, T-BIL, total bilirubin, T-CHO, total cholesterol, TP, total proteins, γ-GT, γ-Glutamyl transpeptidase

## Abstract

The fish paste product, fish balls ‘tsumire’, is a traditional type of Japanese food made from minced fish as well as imitation crab, kamaboko and hanpen. Although tsumire is known as a high-protein and low-fat food, there is a lack of scientific evidence on its health benefits. Hence, we aimed to investigate the effects of tsumire intake on organ weight and biomarker levels in Sprague–Dawley rats for 84 d as a preliminary study. Six-week-old male Sprague–Dawley rats were divided into two groups: group I, fed normal diets, and group II, fed normal diets with 5 % dried tsumire. Throughout the administration period, we monitored their body weight and food intake; at the end of this period, we measured their organ weight and analysed their blood biochemistry. No significant differences were observed with respect to body weight, food intake, organ weight and many biochemical parameters between the two groups. It was found that inorganic phosphorus and glucose levels were higher in group II rats than in group I rats. On the other hand, sodium, calcium, amylase and cholinesterase levels were significantly lower in group II than in group I. Interestingly, we found that the levels of aspartate aminotransferase, alanine transaminase, lactate dehydrogenase and leucine aminopeptidase in group II were significantly lower than in group I, and that other liver function parameters of group II tended to be lower than in group I. In conclusion, we consider that the Japanese traditional food, ‘tsumire,’ may be effective as a functional food for human health management worldwide.

## Introduction

In Japan, fish balls ‘tsumire’ are traditional fish paste products prepared from minced fish (surimi), such as imitation crab, ‘kamaboko’ (fish cake) and ‘hanpen’. In general, fish paste products are made from fish muscle proteins, wherein fish meat is minced with salt to solubilise myofibrillar proteins, such as myosin and actin^(1)^. These products are prepared from many fish species, such as pollack, threadfin bream, white croaker, red bigeye, blue shark and pike eel^(2)^. Compared with other fish paste products, fish ball tsumire is mainly prepared from blue-backed fish, such as sardines and mackerels^(3)^.

Blue-backed fish is one of the most important species in seafood processing^(4)^. This fish is common in Japan, and it is estimated that the Japanese diet usually involves the consumption of more blue-backed fish than that of American and European diets^(5)^. Some fish and fish paste products contain bioactive compounds, such as eicosapentaenoic acid (EPA) and docosahexaenoic acid (DHA), rendering fish protein beneficial for human health. These polyunsaturated fatty acids are found in higher amounts in blue-backed fish than in other fish^(6)^. Arai *et al.* showed that fish oils containing EPA and DHA inhibited body weight gain and exhibited an anti-obesity effect in female KK mice^(7)^. Hung *et al.* reported that serum cholesterol (CHO), triacylglycerol (TAG) and phospholipid levels of Sprague–Dawley rats fed EPA or DHA for 3 weeks were significantly lower than those of the safflower oil–fed rats^(8)^. In addition, a study demonstrated that fish protein hydrolysate decreased the level of plasma total cholesterol (T-CHO) and increased the proportion of high-density lipoprotein cholesterol (HDL-C) in male Wistar rats^(9)^. Moreover, Mizushige *et al.* investigated the effect of Alaska pollack protein (APP) intake with high-fat diet on rats for 4 weeks, wherein they reported that this intake decreased serum TAG and inhibited the accumulation of visceral body fat in the animal^(10)^.

Consequently, we have been persistently determining the effects of fish paste products and/or other products on human health^(11,12)^. In our previous report, we demonstrated the effect of the fish paste product ‘hanpen’ intake on organ weight and biomarker levels in Sprague–Dawley rats fed a diet comprising hanpen for 84 d. We reported that the levels of T-CHO and HDL-C were significantly higher in the hanpen-intake group than in the control group (without hanpen). Conversely, the lactate dehydrogenase (LDH) level was marked lower in the hanpen-intake group than in the control group^(13)^. Tsumire is known as a high-protein and low-fat food in Japan, and the levels of EPA and DHA in tsumire are higher than those in hanpen. Therefore, we can expect that fish balls ‘tsumire’ can have beneficial effects on health. However, there is little literature available that reports its health benefits.

Hence, we aimed to investigate the effects of the fish paste products ‘tsumire’ intake on organ weight and biomarker levels in Sprague–Dawley rats fed a diet comprising tsumire for 84 d in this study.

## Experimental methods

### Materials

**Fig. 1.**
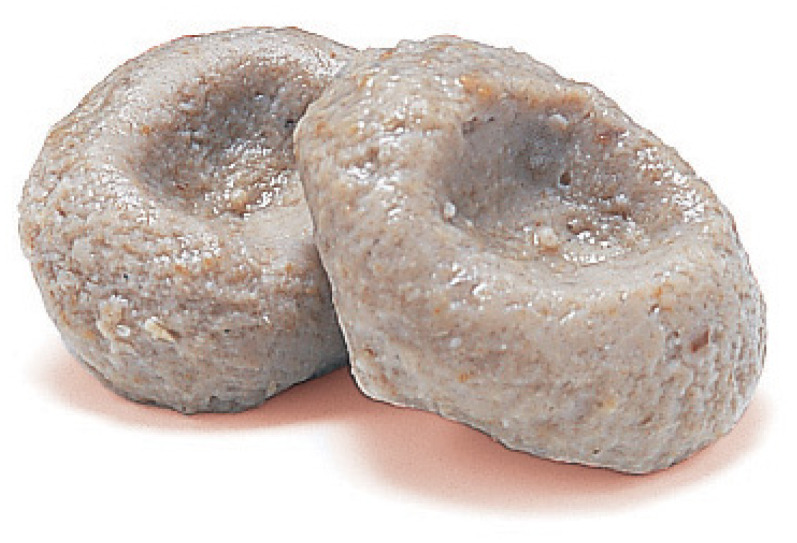
Fish paste product KIBUN tsumire.

Commercial KIBUN tsumire (Fig. 1) was lyophilised. In brief, minced fish, surimi (20 % sardine, 5 % horse mackerel, 15 % pollack and 15 % blue shark), were ground with starch, salt, plant protein, egg white and other ingredients. Mixed fish paste was shaped into a ball-like formation (35 mm × 45 mm) and boiled at 85 °C for 15 min. The breaking strength and breaking strain of this fish ball, KIBUN tsumire, are approximately 170 g and 4·0 cm, respectively.

### Animal studies

Animal experiments were performed as described in our previous report but with a slight modification^(13)^. Six-week-old male Sprague–Dawley rats, purchased from SLC Japan, Inc. (Shizuoka, Japan), were individually housed under a 12/12 h light/dark cycle (light phase; 8·00–20·00, dark phase; 20·00–8·00) at a temperature of 23 ± 5 °C and relative humidity of 55 ± 25 %, with aseptic food and tap water *ad libitum*. The amounts of dried tsumire in experimental diets were similar to those described in our previous report^(13)^. The subjects were divided into two groups: group I, fed AIN-93G (*n* 8), and group II, fed AIN-93G with 5 % dried tsumire (*n* 8); they had free access to food for 84 d. The body weight and food intake of each rat were measured once a week. After 84 d of administration, all rats were sacrificed using isoflurane anaesthesia (the concentration for the induction of anaesthesia: 4 %, concentration for maintenance: 2 %), and a blood sample corresponding to a non-fasting state was collected. The blood samples were centrifuged at 2851 *g* at 4 °C for 10 min and stored at −80 °C until further analyses. The weight of the liver, spleen, kidneys, white adipose tissues, interscapular brown adipose tissues and skeletal muscles of each rat was measured. We calculated the relative organ to body weight (%) in some cases.

The experiments were performed at Kitayama Labes Co., Ltd. in Oriental Yeast Co., Ltd. and authorised by the Japanese Government. The present study was conducted according to the ethical guidelines for laboratory animals and the standard operating procedures of the laboratory. The experimental protocol was approved by the animal experiment ethics committee of the laboratory (approval no. IBC57-049).

### Experimental diets

Experimental diets were prepared as described in our previous report but with a slight modification^(13)^. Rats in group I were fed AIN-93G (Oriental Yeast Co., Ltd., Tokyo, Japan) as the control diet. On the other hand, rats in group II were given a diet wherein dried tsumire replaced casein, l-cystine and β-cornstarch from AIN-93G. The formulation and the nutritional composition of the experimental diets used in this study are shown in Tables 1 and 2, respectively.

**Table 1.** Composition of the experimental diets

	Group I (without tsumire)	Group II (with tsumire)
Casein^a^	20·0	17·7
l-cystine	0·30	0·26
β-Cornstarch	39·7486	35·0886
α-Cornstarch	13·2	13·2
Sucrose	10·0	10·0
Soybean oil	7·0	6·9
Cellulose	5·0	5·0
Mineral mix, AIN-93G-MX^b^	3·5	–
Modified mineral mix	–	3·5
Calcium carbonate	–	1·2
Monopotassium phosphate	–	0·7
Tripotassium citrate	–	0·2
Vitamin mix, AIN-93-VX^c^	1·0	1·0
Choline bitartrate	0·25	0·25
Butylhydroquinone	0·0014	0·0014
Tsumire dry powder	–	5·0
Total (%)	100	100

Experimental diets were prepared as described in our previous report but with a slight modification^(13)^.

a Casein are from milk.

b Mineral mix, AIN-93G-MX contains CaCO_3_, KH_2_PO_4_, K_3_C_6_H_5_O_7_·H_2_O, NaCl, K_2_SO_4_, MgO, FeC_6_H_5_O_7_·XH_2_O, ZnCO_3_, MnCO_3_, CuCO_3_ Cu(OH)_2_·H_2_O, KIO_3_, Na_2_SeO_4_, (NH_4_)Mo_7_O_24_·4H_2_O, Na_2_SiO_3_·9H_2_O, CrK(SO_4_)·12H_2_O, LiCl, H_3_BO_3_, NaF, NiCO_3_ 2Ni(OH)_2_·4H_2_O, NH_4_VO_3_ and sucrose.

c Vitamin mix, AIN-93-VX contains nicotinic acid, potassium pantothenate, pyridoxine hydrochloride, thiamin hydrochloride, riboflavin, folic acid, D-biotin, vitamin B_12_, vitamin E, vitamin A, vitamin D_3_ (cholecalciferol), vitamin K_1_ (phylloquinone) and sucrose.

**Table 2.** Nutritional compositions of the experimental diets

	Group I (without tsumire)	Group II (with tsumire)
Water (g)	9·00	9·00
Energy (kcal)	368·00	372·90
Crude protein (g)	18·10	18·10
Crude fat (g)	7·30	7·30
Crude ash (g)	3·10	1·90
Crude fibre (g)	5·00	5·00
Nitrogen-free extract (g)	57·60	58·70
Ca (g)	0·52	0·52
P (g)	0·32	0·32
Mg (g)	0·05	0·05
Na (g)	0·10	0·14
K (g)	0·36	0·36

Ca, calcium; P, phosphorus; Mg, magnesium; Na, sodium; K, potassium.

Experimental diets were prepared as described in our previous report but with a slight modification^(13)^.

### Measurement of serum biochemical parameters

The collected blood samples were stored at −80 °C until further analyses. Serum biochemical parameters were measured using commercial diagnostic kits and 7180 Clinical Analyzer (Hitachi, Ltd., Tokyo, Japan). Total proteins (TP) were measured using the TP-HR II kit (FUJIFILM Wako Pure Chemical Corporation, Osaka, Japan). Albumin (ALB) was analysed using the ALB II HA-Test Wako kit (FUJIFILM Wako Pure Chemical Corporation, Osaka, Japan). Blood urea nitrogen (BUN) was evaluated using UN-S regents (Denka Company, Ltd., Tokyo, Japan). Creatinine (CRE) was measured using the L-type Wako CRE M kit (FUJIFILM Wako Pure Chemical Corporation, Osaka, Japan). Sodium (Na), potassium (K) and chloride (Cl) were analysed using iron electrode reagents (FUJIFILM Wako Pure Chemical Corporation, Osaka, Japan). Calcium (Ca) was evaluated using Accuras Auto Ca II (Shino-Test Corporation, Tokyo, Japan). Inorganic phosphorus (IP) was measured using Determiner L IP II (Minaris Medical Corporation, Ltd., Tokyo, Japan). Iron (Fe) was analysed using Quick Auto Neo Fe (Shino-Test Corporation, Tokyo, Japan). Aspartate aminotransferase (AST) was evaluated using the L-type Wako AST J2 kit (FUJIFILM Wako Pure Chemical Corporation, Osaka, Japan). Alanine transaminase (ALT) was evaluated using the L-type Wako ALT J2 kit (FUJIFILM Wako Pure Chemical Corporation, Osaka, Japan). Alkaline phosphatase (ALP) was evaluated using the L-type Wako ALP J2 kit (FUJIFILM Wako Pure Chemical Corporation, Osaka, Japan). LDH was evaluated using the L-type Wako LD J kit (FUJIFILM Wako Pure Chemical Corporation, Osaka, Japan). Leucine aminopeptidase (LAP) was measured by using Iatro-LQ LAP rate II (LSI Medience Corporation, Tokyo, Japan). Amylase (AMY) was analysed by using L-type Wako amylase (FUJIFILM Wako Pure Chemical Corporation, Osaka, Japan). γ-Glutamyl transpeptidase (γ-GT) was evaluated using L-type Wako γ-GT J (FUJIFILM Wako Pure Chemical Corporation, Osaka, Japan). Cholinesterase (ChE) was measured using Quick Auto Neo ChE (Shino-Test Corporation, Tokyo, Japan). T-CHO was analysed using L-type Wako CHO M (FUJIFILM Wako Pure Chemical Corporation, Osaka, Japan). Free cholesterol (F-CHO) was evaluated using L-type Wako free cholesterol (FUJIFILM Wako Pure Chemical Corporation, Osaka, Japan). TAG was measured using L-type Wako TG M (FUJIFILM Wako Pure Chemical Corporation, Osaka, Japan). Low-density lipoprotein cholesterol (LDL-C) was analysed using Cholestest LDL (Sekisui Medical Co., Ltd., Tokyo, Japan). HDL-C was evaluated using Cholestest N HDL (Sekisui Medical Co., Ltd., Tokyo, Japan). Glucose (GLU) was measured using Quick Auto Neo GLU-HK (Shino-Test Corporation, Tokyo, Japan). Total bilirubin (T-BIL) was analysed using Nescote VL T-Bill (Alfresa Pharma Corporation, Osaka, Japan). Direct bilirubin (D-BIL) was evaluated using Nescote VL D-Bill (Alfresa Pharma Corporation, Osaka, Japan). Total bile acids (TBA) were measured using Aqua-auto Kainos TBA (Kainos Laboratories Inc., Tokyo, Japan).

### Statistical analysis

The results are presented as mean ± standard error. Statistical significance was evaluated using Student's *t*-test. A *P*-value of less than 0·05 was considered statistically significant. The sample size was calculated by the methods described by Charan and coworkers^(14)^.
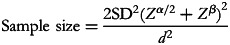
where sd is the standard deviation, *Z^α^*^/2^ is 1·96 at type 1 error of 5 %, *Z^β^* is 0·842 at 80 % power and *d* is the difference between mean values.

The calculated sample size of 7·851 rats per each group was determined based on the efficacy of KIBUN hanpen in influencing liver weights described by our previous experiment^(13)^. So, we chose eight rats in each group in this study.

## Results

### Effect of tsumire intake on body weight, organ weight, adipose tissue weight and muscle weight in Sprague–Dawley rats

Table 3 is summarised as described in our previous report^(13)^. The effects of the oral administration of AIN-93G with 5 % dried tsumire on the body weight, organ weight, adipose tissue weight and muscle weight in Sprague–Dawley rats are summarised in Table 3. Briefly, the differences in food intake, body weight, liver weight, spleen weight, adipose tissue weight and muscle weight between the two groups were not significant after administration of the experimental diets for 84 d. On the contrary, the kidney weights of group II (3·08 ± 0·10 g) were higher than those of group I (2·74 ± 0·08 g).

**Table 3.** Effect of tsumire intake on body weight, organ weight, adipose tissue weight and muscle weight in Sprague–Dawley rats after 84 d of administration (*n* 8 in each group)

	Group I (without tsumire)	Group II (with tsumire)
Total food intake (g)	1669·00 ± 40·64	1737·63 ± 32·82
Initial body weight (g)	206·40 ± 2·23	206·00 ± 2·00
Final body weight (g)	528·30 ± 15·93	539·80 ± 9·76
Liver (g)	17·49 ± 0·47	17·98 ± 0·84
Kidneys (g)	2·74 ± 0·08	3·08 ± 0·10*
Spleen (g)	0·88 ± 0·03	0·89 ± 0·03
White adipose tissue (g)	34·29 ± 1·31	33·22 ± 1·75
Perirenal (g)	15·32 ± 0·70	14·18 ± 0·51
Epididymal (g)	12·94 ± 0·48	13·18 ± 0·92
Mesenteric (g)	6·02 ± 0·45	5·86 ± 0·49
Brown adipose tissue (g)	0·64 ± 0·08	0·70 ± 0·07
Muscle (g)	6·63 ± 0·23	6·66 ± 0·23
Soleus (g)	0·41 ± 0·02	0·41 ± 0·01
Gastrocnemius (g)	6·22 ± 0·21	6·25 ± 0·22

Results are expressed as mean ± standard error. Statistical significance is evaluated by Student's *t*-test.

* *P* < 0·05 *v*. Group I.

Table 3 is summarised as described in our previous report^(13)^.

### Effect of tsumire intake on biochemical parameters in Sprague–Dawley rats

Table 4 is summarised as described in our previous report^(13)^. Table 4 shows an analysis of the blood biochemical parameters of rats after administration of the diets containing 5 % dried tsumire. No significant differences were seen in almost all the biochemical parameters between the two groups. However, the levels of IP and the GLU were higher in group II than in group I. On the other hand, Na, Ca, AMY and ChE levels were significantly lower in group II than in group I.

**Table 4.** Effect of tsumire intake on blood biochemical parameters in Sprague–Dawley rats after 84 d of administration

	‘0’ d administration (*n* 8)	84 d administration (*n* 8)	
		Group I (without tsumire)	Group II (with tsumire)
TP (g/dl)	6·36 ± 0·05	6·55 ± 0·09	6·40 ± 0·13
ALB (g/dl)	4·61 ± 0·03	4·38 ± 0·04	4·15 ± 0·09
A/G (−)	2·65 ± 0·06	2·03 ± 0·07	1·85 ± 0·05
BUN (mg/dl)	19·73 ± 1·34	19·40 ± 0·66	17·91 ± 0·31
CRE (mg/dl)	0·23 ± 0·01	0·36 ± 0·01	0·36 ± 0·02
Na (mEq/l)	141·63 ± 0·17	142·13 ± 0·37	140·13 ± 0·41**
K (mEq/l)	5·03 ± 0·09	4·48 ± 0·03	4·65 ± 0·12
Cl (mEq/l)	100·88 ± 0·45	101·13 ± 0·33	101·00 ± 0·25
Ca (mg/dl)	11·46 ± 0·10	10·64 ± 0·08	10·38 ± 0·06*
IP (mg/dl)	8·74 ± 0·24	5·70 ± 0·08	6·20 ± 0·15*
Fe (μg/dl)	208·25 ± 9·20	162·50 ± 5·33	156·00 ± 9·12
AST (IU/l)	118·88 ± 10·26	104·50 ± 3·36	76·25 ± 2·75**
ALT (IU/l)	43·50 ± 2·11	30·13 ± 1·39	26·13 ± 1·05*
ALP (IU/l)	1865·75 ± 220·78	499·75 ± 56·04	396·50 ± 32·61
LDH (IU/l)	1021·13 ± 165·60	1521·25 ± 72·75	952·50 ± 131·97**
LAP (IU/l)	60·38 ± 1·67	55·00 ± 1·51	49·63 ± 1·07*
AMY (IU/l)	2577·75 ± 123·15	2065·50 ± 76·41	1839·38 ± 57·05*
γ-GT (IU/l)	<3·00	<3·00	<3·00
ChE (IU/l)	13·13 ± 1·02	9·75 ± 0·34	6·63 ± 0·43**
T-CHO (mg/dl)	96·13 ± 4·27	95·00 ± 4·07	87·13 ± 6·59
F-CHO (mg/dl)	20·63 ± 1·09	15·75 ± 0·86	16·38 ± 1·53
E-CHO (mg/dl)	75·50 ± 3·23	79·25 ± 3·30	70·75 ± 5·12
E/T (%)	78·63 ± 0·30	83·38 ± 0·39	81·25 ± 0·55*
TAG (mg/dl)	113·38 ± 7·96	133·50 ± 11·95	135·25 ± 26·40
LDL-C (mg/dl)	11·63 ± 0·73	9·63 ± 0·56	8·13 ± 0·67
HDL-C (mg/dl)	40·25 ± 1·20	32·75 ± 0·82	30·38 ± 1·75
AIP^a^ (–)	0·08 ± 0·03	0·16 ± 0·05	0·23 ± 0·09
GLU (mg/dl)	135·00 ± 1·59	158·63 ± 7·60	230·13 ± 14·25**
T-BIL (mg/dl)	0·04 ± 0·00	0·06 ± 0·00	0·08 ± 0·01
D-BIL (mg/dl)	0·01 ± 0·00	0·03 ± 0·00	0·03 ± 0·00
I-BIL (mg/dl)	0·02 ± 0·00	0·04 ± 0·00	0·05 ± 0·01
TBA (μmol/l)	21·63 ± 1·85	13·88 ± 2·25	11·38 ± 2·13

TP, total proteins; ALB, albumin; BUN, blood urea nitrogen; CRE, creatinine; Na, sodium; K, potassium; Cl, chloride; Ca, calcium; IP, inorganic phosphorus; Fe, iron; AST, aspartate aminotransferase; ALT, alanine transaminase; ALP, alkaline phosphatase; LDH, lactate dehydrogenase; LAP, leucine aminopeptidase; AMY, amylase; γ-GT, γ-glutamyl transpeptidase; ChE, cholinesterase; T-CHO, total cholesterol; F-CHO, free cholesterol; E-CHO, esterified cholesterol; TAG, triacylglycerols; LDL-C, low-density lipoprotein cholesterol; HDL-C, high-density lipoprotein cholesterol; AIP, atherogenic index of plasma; GLU, glucose; T-BIL, total bilirubin; D-BIL, direct bilirubin; I-BIL, indirect bilirubin; TBA, total bile acids.

Results are expressed as mean ± standard error. Statistical significance is evaluated by Student's *t*-test.

** *P* < 0·01 *v*. Group I, *P < 0·05 *v*. Group I.

a AIP: log (TAG/HDL-C) with TAG (mg/dl/88·57) and HDL-C (mg/dl/38·67) expressed in mmol/l ^(15)^.

Table 4 is summarised as described in our previous report^(13)^.

The levels of AST, ALT, ALP, LDH and LAP of groups I and II are shown in Table 4. The levels were significantly lower in group II than in group I.

## Discussion

The traditional Japanese diet includes rice, miso soup, soybean products, vegetables, fruits, Japanese pickles, seaweed, mushrooms, green tea and fish^(16^^)^. Such a traditional diet along with other Japanese food products have been widely known to be healthy, contributing to longevity and prevention of various noncommunicable diseases^(17,18)^. Further, fish paste products, such as kamaboko, imitation crab, hanpen and tsumire, are traditional and commonly consumed foods in Japan. These fish paste products are prepared from minced fish, starch and salt; they are easy to eat as compared with raw fish that contains skeletal bones and fish guts. The characterisation of fish balls tsumire is that they are made from mainly blue-backed fish, such as sardines and mackerels,^(3)^ as compared with other fish paste products, and blue-backed fish-based surimi fish paste is shaped into balls and boiled (Fig. 1). The tsumire that we used in the present study (KIBUN tsumire) contains approximately 11·9 % protein and 1·8 % fat. Moreover, the EPA and DHA values of this commercial tsumire (per 100 g) are 170 and 310 mg, respectively. In our previous study, we reported the effect of the fish paste product ‘hanpen’ intake on organ weight and biomarker levels in Sprague–Dawley rats fed a diet comprising hanpen for 84 d^(13)^. However, few studies have reported the health benefits of the fish balls tsumire. Against this background, in this study, we investigated the effects of tsumire intake on organ weight and biomarker levels in Sprague–Dawley rats for the first time.

We did not observe any fatalities or abnormalities with respect to food consumption and coat condition in the tsumire-administered rats (group II). From these findings, we suggest that tsumire did not induce any adverse reaction in rats after 84 d of administration. No significant differences were found in the total food intake, body weight, organ weight and several biochemical parameters between groups I and II since the nutrition levels of both groups were similar (Table 2).

We observed kidney weight gains in group II unlike in group I (*P* < 0·05) after the administration period. Subsequently, we calculated the relative organ to body weight (%) using the formula described by Vani and Reddy^(19)^ and our previous report^(13)^.
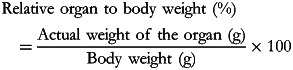
The relative organ to body weight of kidney was 0·57 % in group II, which was not significantly different from that of group I (0·52 %). Additionally, there were no significant differences in the final body weight, liver weight, spleen weight and adipose tissue weight (white and brown) between the two groups. Moreover, there were no differences in muscle weight (soleus and gastrocnemius muscles) between the groups. Group I diet, AIN-93G, contained 20 % casein as the main protein source, whereas group II diet contained 5 % dried tsumire that mainly replaced casein. Reidy *et al.* reported that protein ingestion in the form of casein and whey increases the supply of amino acids to muscles, which further promotes muscle protein synthesis^(20)^. In addition, cod protein exhibited anti-inflammatory activity and has an effect on skeletal muscle repair after an injury in male Wistar rats^(21)^. As a result of our findings, which are concurrent with the aforementioned reports, we consider that fish balls tsumire is a protein source for skeletal muscle synthesis as well as casein. In our previous report, we showed that fish paste products hanpen is a potential protein source for skeletal muscle synthesis as well as casein and tsumire. Therefore, we suggest that the fish paste products, tsumire and hanpen, are protein sources for muscle synthesis in human subjects. Particularly, dried tsumire consists of approximately 46, 3·5, 2·1 and 2·3 % protein, leucine, isoleucine and valine residues, respectively. These three residues are essential and fall under the category of branched-chain amino acids (BCAAs). BCAAs are widely popular, whereby their intake combined with resistance physical exercise stimulates muscle protein synthesis^(22)^.

Blood biochemistry analysis showed that the IP level was higher in group II than in group I (*P* < 0·05). However, concentrations of Na and Ca were lower in group II than in the other group (Na: *P* < 0·01, Ca: *P* < 0·05). Hatayama *et al.* investigated the background data of blood biochemistry parameters in Crj:CD(sd)IGS rats^(23)^. They reported that the standard value of IP is 7·7 ± 0·6, 6·5 ± 0·8 and 6·0 ± 0·9 mg/dl; that of Na is 143 ± 1, 143 ± 2 and 144 ± 2 mg/dl and that of Ca is 9·9 ± 0·3, 10·1 ± 0·4 and 10·2 ± 0·3 mg/dl, in 10-, 19- and 32-week-old rats, respectively. Consequently, we consider that the IP, Na and Ca levels of group II rats (with tsumire) were almost equivalent to these standard values.

Liver is a vital organ that carries out essential functions, including detoxification of deleterious materials, regulation of numerous metabolic functions and maintaining body homeostasis^(24)^. Serum AST, ALT, ALP, LDH and LAP levels are well-known clinical markers of liver damage^(25)^. In this study, the AST, ALT, LDH and LAP levels of group II (with tsumire) were significantly lower than those of group I (without tsumire), and the ALP level of group II tended to be lower than that of group I. These data suggest that tsumire intake may be effective in preventing liver function deterioration. Polyunsaturated fatty acids, including EPA and DHA, are recommended as a dietary strategy against non-alcoholic fatty liver disease, since they exhibit high-antioxidant, anti-inflammatory and hypolipidemic effects^(26,27)^. Most studies on EPA- and DHA-containing fish oil have focused on the prevention and treatment of cholestasis^(28,29)^. In addition, fish oil has been used in the treatment of intestinal failure–associated liver disease since the 2000s^(30)^. Further, Zhang *et al.* reported that fish oil was effective in children with intestinal failure–associated liver disease and improved liver function, as demonstrated by ALT and AST levels^(31)^. Consequently, we suggest that the polyunsaturated fatty acids, including EPA and DHA, that are reportedly present in KIBUN tsumire, contributed to a lowering of the level/concentration of liver function–related enzymes in group II. Previously, we have reported that only LDH level was significantly lower in the group administered with the fish paste product hanpen as compared with that in the control group (without hanpen) in Sprague–Dawley rats after 84 d of administration^(13)^. This is despite the fact that tsumire intake was more potent than that of hanpen for preventing liver function deterioration, because the DHA and EPA levels of dried tsumire were 1·3 and 0·65 %, respectively, which were 8-fold and 6-fold, respectively, than those of dried hanpen.

AMY is an exocrine enzyme secreted by the pancreas and salivary glands. Serum AMY has been used to provide diagnostic support for acute and chronic pancreatitis in clinical practice^(32)^. In general, serum AMY levels of people with pancreatitis are higher than those of people without pancreatitis^(33)^. In this study, we observed that the AMY levels of group II (with tsumire) were significantly lower those that of group I (without tsumire). Therefore, we suggest that tsumire intake is effective in preventing pancreas function deterioration. Alhan *et al.* reported that polyunsaturated fatty acids, such as EPA and DHA, have a beneficial effect on a wide range of chronic and acute pancreatitis^(34)^.

Further, compared with the other group, ChE levels were significantly lower in group II (with tsumire). ChEs are a group of enzymes that hydrolyse acetylcholine and other choline esters. They are two categorised into two, namely, acetylcholinesterase and butyrylcholinesterase (BChE)^(35)^. BChE activity in human subjects is related to the hepatobiliary disease, hyperlipidemia, diabetes and cardiovascular disease in adult patients^(36)^. Moreover, there was a report that BChE levels of Alzheimer's-like disease model rats were significantly higher than those of control rats^(37)^.

Contrary to our expectations, we observed that the GLU level of group II was higher than that of group I. However, we assume that this result does not signify any problem because no deaths or abnormalities in food consumption and coat condition were observed in the tsumire-administered rats in this study. Previously, a few studies have demonstrated that omega-3 fatty acids, such as α-linolenic acid (ALA) and DHA, increase plasma glucagon-like peptide-1 (GLP-1) levels *in vivo*^(38,39)^. On the contrary, a report mentions that GLP-1 and blood GLU levels are unaffected by omega-3 fatty acid supplementation in Sprague–Dawley rats exists as well^(40)^. In all, the mechanisms underlying the increase of blood GLU levels due to tsumire remain unclear, which demands further research in this area.

In conclusion, the commercially available Japanese traditional food, KIBUN tsumire, facilitated skeletal muscle synthesis as well as casein, and prevention of liver and pancreas function deterioration after 84 d of administration in Sprague–Dawley rats. Moreover, tsumire intake was more potent than that of hanpen^(13)^ for preventing liver function deterioration. Tsumire is easy to eat in comparison with raw fish, which contains skeletal bones and fish guts that are similar to other fish paste products^(13)^, and is considered beneficial to human health. The present study is a preliminary investigation of the effects of tsumire intake on organ weight and biomarker levels in rats. However, the mechanisms by which tsumire aids in preventing liver and pancreas function deterioration remain unclear. In addition, the differential effects of tsumire intake among young and aged animals need to be studied. In summary, we strongly consider that tsumire can be an effective functional food for human health management worldwide, in particular, skeletal muscle synthesis and prevention of liver and pancreas function deterioration.
